# HyperArc VMAT stereotactic radiotherapy for locally recurrent previously‐irradiated head and neck cancers: Plan quality, treatment delivery accuracy, and efficiency

**DOI:** 10.1002/acm2.13561

**Published:** 2022-03-07

**Authors:** Damodar Pokhrel, Mark E Bernard, Jeremiah Johnson, Denise Fabian, Mahesh Kudrimoti

**Affiliations:** ^1^ Medical Physics Graduate Program Department of Radiation Medicine University of Kentucky Lexington Kentucky USA

**Keywords:** Delivery efficiency, hyperArc VMAT, recurrent, head and neck SRT, treatment option

## Abstract

**Purpose:**

This paper demonstrates the clinical feasibility and efficacy of HyperArc VMAT treatments for locally recurrent, locally advanced, or previously irradiated head and neck cancers treated with stereotactic radiotherapy (SRT).

**Materials/Methods:**

First, an anthropomorphic SRS head phantom from the MD Anderson's IROC credentialing laboratory containing a 1.9 cm diameter spherical target, including in vivo dosimetry system, was imaged, planned, and irradiated (25 Gy in 1 fraction) using HyperArc VMAT with a 6 MV flattening filter free (FFF) beam. Second, RANDO phantom was imaged, planned, and irradiated (35 Gy in 5 fractions) by generating eight HyperArc VMAT plans (4 right, 4 left neck tumors) at different anatomical locations (C1–C4). Average tumor volume was 21.7 cm^3^ up to 32.3 cm^3^. Distance to isocenter from the central marker of the Encompass device down to neck was 25.8 cm up to 28.0 cm and 24.3 cm up to 27.1 cm for left‐ and right‐sided neck tumors, respectively, and 9 cm from both lateral markers defined by the patient protection zone. Third, seven recurrent head and neck cancer patients with 80.3 cm^3^ tumors on average, and up to 159 cm^3^, were imaged, planned, and treated with 30–40 Gy in 5 fractions with HyperArc SRT. Plan quality, treatment delivery accuracy, and efficiency are reported herein.

**Results:**

Phantom irradiation results met all the compliance requirements set forth by the IROC for HyperArc SRS treatment. For end‐to‐end RANDO phantom tests, a highly conformal target dose distribution with 50% isodose fall‐off within 5 mm from the surface of the target was obtained. Average beam modulation factor, beam‐on‐time, and overall treatment time were 2.9, 2.56 min, and 13.96 min with 99.1% pre‐treatment quality assurance pass rate for the 2%/2 mm gamma criteria, respectively. Immediately adjacent critical structures, such as the spinal cord (maximum, 3.9 Gy and 0.35 cm^3^ of cord, 3.7 Gy) and skin (maximum, 10.3 Gy and 10 cm^3^ of skin, 5.7 Gy), were spared. Similar results were found on the patient's HyperArc VMAT plans including highly conformal target coverage, sharp dose fall‐off, and low doses to the adjacent critical organs such as the spinal cord (< 5 Gy). Average perfect pitch couch correction was <1.5 mm and 2° in each direction. Average beam‐on‐time was approximately 3.21 min and treatments were completed within 15 min.

**Conclusion:**

For recurrent head and neck SRT treatments, HyperArc VMAT provided highly conformal dose distributions, rapid dose fall‐off, excellent sparing of adjacent critical organs, and highly accurate treatments that could be delivered down to the C4 vertebral level. This could potentially allow for delivery of HyperArc SRT to patients with glomus tumors as well to those who may not tolerate frame‐based SRS treatment. Clinical follow up of these patients is ongoing to confirm the therapeutic benefits of this novel treatment option.

## 1 INTRODUCTION

Over the past few decades, stereotactic radiosurgery/radiotherapy (SRS/SRT) treatment of skull base tumors has demonstrated durable tumor control and symptomatic relief with acceptable toxicity in patients with malignant tumors. Many researchers have described the utilization of fractionated SRT for re‐irradiation of head and neck tumors, primarily using CyberKnife radiosurgery.^1^, ^2^, ^3^, ^4^, ^5^, ^6^, ^7^, ^8^ Some literature exists regarding the use of Linac‐based SRT for head and neck recurrent cancers.^8^, ^9^, ^10^, ^11^, ^12^ For instance, Unger and his colleagues presented a feasibility study concerning re‐irradiating head and neck cancer patients using the CyberKnife technique with fractionated‐SRT scheme.^2^ For 65 patients, the median initial radiation dose was 67 Gy (in 33 fractions), and the median re‐irradiation SRT dose was 30 Gy (21–35 Gy) in 2–5 fractions. In their study, the median follow‐up for surviving patients was 16 months. Out of 56 patients that were evaluated for radiation response, 30 patients (54%) had shown complete response. However, seven patients (11%) experienced severe re‐irradiation related toxicity. Siddiqui and colleagues studied the feasibility, safety, and efficacy of Linac‐based SBRT in patients with primary, recurrent, and metastatic head and neck tumors.^11^ Fifty‐five tumors in 44 patients were treated with either a single‐fraction of 13–18 Gy or 5–8 fractions schemata to a total dose of 36–48 Gy. For the 24 patients who had clinical follow‐up scans, the percentage of reduction in tumor volume was 52%± 38%. Local control rate at 1 year was 83.3% for primary tumors and 60.6% for recurrent tumors. The University of Pittsburgh experience, which consisted primarily of robotic CyberKnife treatments, suggests a trend of improved dose response and survival with doses of 40 Gy or higher.^8^ However, due to major concerns with patient collision and delivery efficiency, these early Linac‐based studies primarily used coplanar beam and arc geometry for recurrent head and neck SRT patients.

Recent technological advancements in the delivery of the Linac‐based SRS/SRT treatment, including utilization of highly reproducible patient positioning and image‐guided localization procedures, have become a viable treatment modality for inoperable patients with primary, recurrent, or metastatic skull‐based tumors including head and neck cancers. For example, Varian TrueBeam version 2.7 or higher Linac (Varian Medical Systems, Palo Alto, CA) configured with recently utilized a fully automated highly conformal non‐coplanar‐arc geometry with the Eclipse Treatment Planning System (TPS, Eclipse, version 15.1 or higher) as the HyperArc VMAT module for the treatment of multiple intracranial lesions, and this development has generated global clinical interest.^13^, ^14^, ^15^, ^16^, ^17^, ^18^, ^19^ In addition to fast treatment delivery, our current clinical experience with the HyperArc VMAT module has provided highly conformal dose distributions to the cranial lesions with steep dose gradients, which has the potential to spare the adjacent critical organs and to improve patient set up and target localization accuracy. We therefore utilized the novel application of the non‐coplanar HyperArc VMAT for recurrent head and neck cancer patients to provide a more highly conformal dose distribution, while sparing adjacent critical organs, such as the spinal cord. In this paper, we demonstrate the independent validation and initial clinical implementation of the HyperArc VMAT for SRT treatments of recurrent head and neck cancer patients that utilize the built‐in patient protection zone and avoids the patient collision issue.

## 2 METHODS AND MATERIALS

### 2.1 Credentialing HyperArc VMAT stereotactic radiosurgery

An independent dose verification of the HyperArc VMAT SRS treatments on the TrueBeam Linac (Varian Medical Systems, Palo Alto, CA) was performed using the IROC MD Anderson's SRS credentialing anthropomorphic head phantom containing a 1.9 cm diameter spherical target and dosimetry systems (two orthogonal films and two thermoluminescent dosimeters, TLD capsules) inserted. This phantom was imaged using the HyperArc set up (Eclipse, Version 15.6, Varian Medical Systems, Palo Alto, CA) with Q‐fix mask, head rest, and the Encompass device, planned and irradiated with an SRS prescription dose of 25.0 Gy in 1 fraction to the target for credentialing based upon the Alliance A071801 brain SRS/SRT trial. A full non‐coplanar HyperArc VMAT geometry with 1 full co‐planar arc and 3‐partial arcs with 6‐MV‐flattening filter free (FFF) beam on TrueBeam Linac equipped with 120Millenium MLC and a perfect pitch couch was used. Advanced Acuros‐based dose calculation was validated in the Varian Eclipse TPS. The credentialing results of the MD Anderson's anthropomorphic thorax phantom incorporating dosimetry inserts in the tumor satisfied both the TLD and film dosimetric requirements established by the IROC for the SRS/SRT treatment on TrueBeam Linac. In this independent measurement, the average TLD and film measurement results were within ±2.0% and 97% gamma index over all three planes, respectively. The phantom irradiation results met the MD Anderson's credentialing requirement.

### 2.2 Phantom's HyperArc VMAT plans

The in‐house RANDO head phantom was imaged, planned, and irradiated (35 Gy in 5 fractions) by generating eight HyperArc VMAT SRT plans (4 right and 4 left‐sided neck tumors) at different anatomical locations (C1–C4). The average tumor volume was 21.7 cm^3^ (13.0–32.3 cm^3^). Distance to isocenter from the central marker of the Encompass device down to the neck was 25.8 cm (23.8–28.0 cm) and 24.3 cm (22.0–27.1 cm) for the left and right‐sided neck tumors, respectively. The lateral distance to the isocenter was 9 cm (inside the patient's anatomy), on average, from the both lateral markers that defined the patient protection zone. For the right‐sided neck tumor a selection of non‐coplanar HyperArc VMAT modules were automatically chosen based on the target location (gantry 0°–180°; couch 0°, 90°, 315°) and for the left‐sided neck tumor (gantry 0°–180°; couch 0°, 270°, 45°), using 6 MV‐FFF beams, while avoiding the beam entrance through the opposite side of the patient anatomy. For full scatter simulation, the body contour was expanded to include the Q‐fix mask, head rest, Integrated Shim^TM^ System, and IntegraBite^TM^. Thus, after completing all the phantom validation testing and measurements, we used HyperArc VMAT SRT for the treatment of recurrent head and neck tumors on our TrueBeam Linac.

### 2.3 Patient's HyperArc VMAT plans

After approval by our institutional review board, seven re‐irradiation patients were identified and included in this study. These patients had been previously treated with conventionally fractionated treatment between 60–70 Gy to the head and neck. For HyperArc VMAT SRT planning, these patients were immobilized using the Q‐fix mask, head rest, and mouth bite with Encompass device (Qfix, Avondale, PA) while lying in the supine position with arms down on the hand grip array. A high‐resolution 3D‐CT imaging was obtained on a GE Lightspeed 16 slice CT scanner (General Electric Medical Systems, Waukesha, WI) with 512 × 512 pixel image size and 1.25 mm slice thickness in the axial helical mode, which included central and lateral markers on the Encompass device and the CT scans with the patient's shoulders. Previously obtained contrast enhanced high‐resolution magnetization‐prepared rapid gradient‐echo (MP‐RAGE) MRI and/or PET/CT images were co‐registered to the planning CT images in the Varian Eclipse TPS for gross tumor volume (GTV) and organs at risk (OAR) delineation. The target volumes were delineated by an experienced radiation oncologist and the GTV was defined by the visible tumor mass. The planning target volume (PTV) was created using a uniform 3.0–5.0 mm expansion margin around each GTV utilizing departmental SRT protocol. The tumor characteristics are summarized in Table 1, which shows wide varieties and sizes of tumor volume with an average PTV of 80.3 ± 55.4 cm^3^ (8.9–159.0 cm^3^). For full scatter simulation, the body contour was expanded (in Eclipse TPS) to include the Q‐fix mask, SRS head rest, Integrated Shim ^TM^ System, and IntegraBite^TM^ mouthpiece. Nearby OARs (spinal cord, skin brainstem, larynx, cochlea including normal brain and optic apparatus) were delineated for dose reporting.

**TABLE 1:** Main tumor characteristics of all seven recurrent head and neck SRT patients included in this study

**Patient #**	**Tumor location**	**GTV (cm^3^)**	**PTV (cm^3^)**	**No. of arcs used, couch positions**
I	Rt ethmoid sinus	116.1	159.0	1‐full, 3 partial arcs (couch: 0°, 45°, 90°, 315 °)
II	Rt base‐of‐skull	28.2	56.2	3 partial arcs (couch: 0°, 90°, 315°)
III	Lt neck mass	35.9	65.3	3 partial arcs (couch: 0°, 270°, 45°)
IV	Lt neck mass	15.2	28.1	3 partial arcs (couch: 0°, 270°, 45°)
V	Rt ethmoid sinus	87.7	130.5	1‐full, 3 partial arcs (couch: 0°, 45°, 90°, 315°)
VI	Rt estomitous sinus	2.7	8.9	1‐full, 3 partial arcs (couch: 0°, 45°, 90°, 315°)
VII	Rt neck mass	63.2	114.0	3 partial arcs (couch: 0°, 90°, 315°)

Each patient was planned prospectively by an experienced physicist using the fully automated non‐coplanar HyperArc VMAT Module in the Varian Eclipse TPS. Doses of 30–40 Gy in 5 fractions were prescribed to 95% of the PTV receiving 100% of the dose. Total dose and fraction size were based mostly on tumor size, volume, location of the tumor, and prior radiation dose with wide varieties of treatment sites and tumor volumes. Other factors, such as proximity of normal tissue organs, and the patient's general condition and tolerability/comorbidity were also considered. Since all the patients received prior radiation therapy, special attention was paid to further optimize the dose distributions with higher priority by sparing the dose to adjacent critical organs including the spinal cord as much as possible (assuming that all previously treated patients received maximal dose of 45 Gy to the spinal cord) and following the head and re‐treatment SRT protocols.^20^, ^21^ Heterogenous target doses were allowed, and the hotspot in the center of the GTV were encouraged to increase the mean dose to GTV. For all HyperArc SRT plans, the isocenter was automatically chosen in the center of the PTV and were still allowable isocenter locations that were within a specific patient protection zone defined by the HyperArc module that reduce the risk of gantry collision with the patient. All HyperArc VMAT plans utilized either one‐full coplanar arc and three‐partial non‐coplanar arcs, or three‐partial arcs which were automatically selected based on tumor location and patient anatomy to avoid the gantry collision (Table 1). The collimator angle for each arc was also automatically optimized based on the target shape and location and beam angles to minimize the leakage dose and improve the target dose conformity.^14^ For HyperArc VMAT SRT planning, the goal was to maintain highly conformal dose distribution to the recurrent target and minimize dose to adjacent critical organs as much as possible, including the spinal cord.^20^, ^21^

### 2.4 Plan evaluation

The dose–volume histograms (DVHs) and isodose curves of HyperArc VMAT SRT plans were evaluated for target conformity, GTV coverage, gradient and heterogeneity indices, and maximal dose 2 cm away from the target (*D2cm*). Using the percentage prescribed isodose volume and target size, the RTOG conformity index (CI) and the Paddick conformation number (PCN) were calculated.^22^, ^23^ The maximal dose to immediately adjacent OAR was recorded for the optic apparatus, brainstem, spinal cord, larynx, mandible, skin, and mean brain dose. Furthermore, treatment delivery efficiency and accuracy were documented by recording the total number of monitor units (MU) per fraction and the ratio of the total number of MU per fraction to the prescription dose in cGy is defined as the modulation factor (MF). The beam‐on time (BOT) was recorded during the treatment delivery of the HyperArc VMAT plan. For each patient, BOT was added to patient set up and verification time, including conebeam CT imaging, image registration, dry‐run, and estimated overall treatment times. Dosimetric verification of these plans was performed using the pre‐treatment patient‐specific quality assurance (QA) with portal dosimetry (PD) measurement following the previously established QA guidelines.^24^ ^,^ ^25^A gamma evaluation criteria of 2%/2 mm with a low‐dose threshold set to 10% was used with > 95% γ‐pass threshold. Moreover, for 2^nd^ physics check, these HyperArc VMAT SRT plans were re‐calculated using an in‐house Monte Carlo (MC) program^26^ that was based on the PENELOPE MC code.^27^ The mean and standard deviation (range) for each dose metric were documented for all dosimetric parameters, target coverage, OAR doses, and treatment delivery parameters. Statistical analysis was performed using the Microsoft Excel (Microsoft Corp, Redmond WA) program.

## 3 RESULTS

### 3.1 RANDO phantom data

**FIGURE 1:**
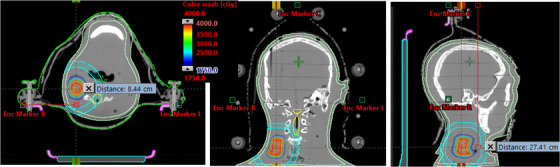
Axial, coronal, and sagittal views of isodose colorwash (50%–115%) for the RANDO phantom's end‐to‐end test HyperArc VMAT (35 Gy in 5 fractions) plan with 3.0 cm diameter tumor in the right neck at the C4 level. Pink and light blue are Encompass devices; skin, body contour, *D*_2cm_ ring and spinal cord are shown. Distance from central marker of the Encompass device to the isocenter (SI‐direction) to right neck tumor was 27.4 cm and lateral distance of 8.5 cm from the right marker—avoiding the patient's collision

Steep dose gradients and faster treatment delivery were achieved with 6 MV‐FFF HyperArc VMAT SRT plans. For the phantom plan shown in Figure 1, the PTV was 13.5 cm^3^ (3.0 cm diameter) and located in the right lower neck at the C4 level. Right‐sided HyperArc geometry of three‐partial arcs of each 180° arc length at 0°, 90°, and 315° couch positions and automatically selected collimator angles were used. This plan provided a total of 1899 MU per fraction with beam MF of 2.9 that was delivered for 35 Gy in 5 fractions. Beam on time was 2.71 min per treatment with overall estimated dose‐to‐door treatment time was less than 15 min. In this case, the HyperArc VMAT SRT plan provided CI, PCN, *D*_2cm_, and GI were 1.06, 0.87, 22.4%, and 2.89, respectively with 6 MV‐FFF beam, all parameters were highly desirable for the head and neck SRT treatment.

For the end‐to‐end tests with the RANDO phantom, the average CI, Paddick conformation number, gradient and heterogeneity indices, and dose to 2 cm away from the target were 1.08 ± 0.05 (1.02–1.14), 0.85 ± 0.02 (0.83–0.89), 2.71 ± 0.27 (2.28–2.92), 0.14 ± 0.02 (0.11–0.17), and 23.5% ± 1.5% (21.4%–25.0%), respectively. Maximal dose to skin and spinal cord was 10.3 ± 3.5 Gy (9.2–14.7 Gy) and 3.9 ± 0.8 Gy (4.0–4.8 Gy), on average. Average dose to 10 cm^3^ of skin was < 5.7 Gy. Mean values of the total MU/fraction, MF, beam on time, and overall treatment time were 2044 ± 378 (1582–2644), 2.96 ± 0.5 (2.39–3.78), 3.1 ± 0.2 min (2.67–3.78 min), and 13.96 ± 0.50 min (13.39–14.78 min), respectively, with no collision issue. The average pre‐treatment portal‐dosimetry QA pass rate was 99% for the 2%2mm gamma criteria.

### 3.2 Patient's plan characteristics

**FIGURE 2:**
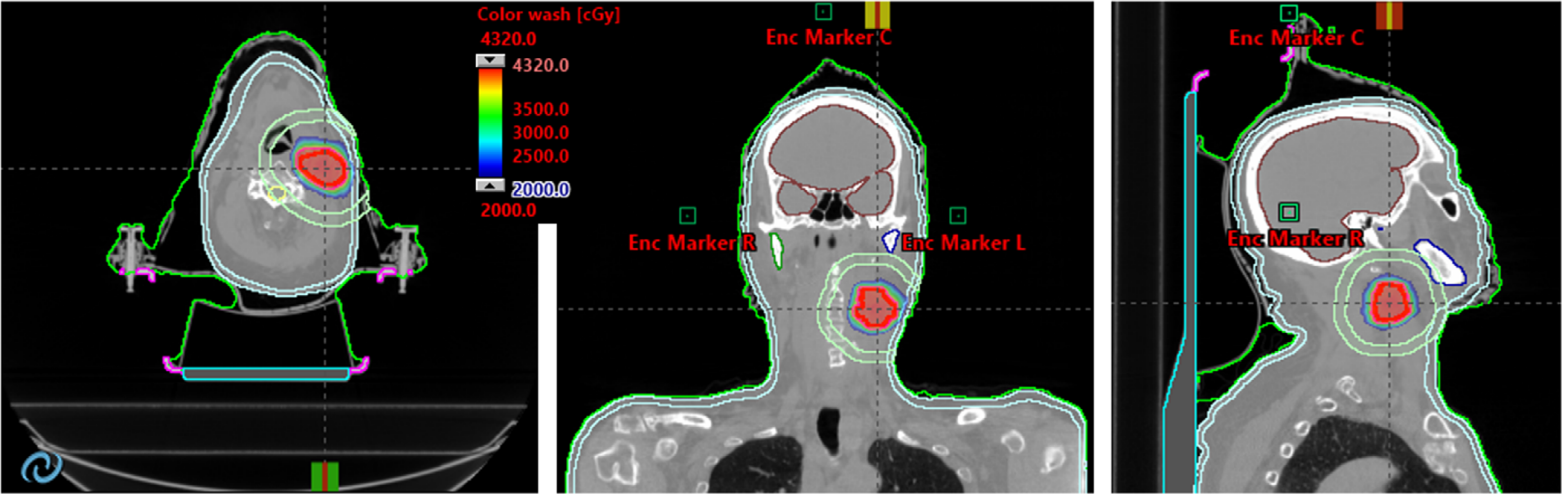
Isodose colorwash distributions for the re‐treatment HyperArc SRT for one of the representative patients in the study (patient #IV). Isodose colorwash is shown for 50% fall‐off within approximately 5 mm from the surface of the PTV. The *D*_2cm_ and critical organs including spinal cord (yellow), mandible (blue), skin (light blue), and brain (brown) contours are shown. Encompass device (pink) and Encompass base (light blue) are also shown

**FIGURE 3:**
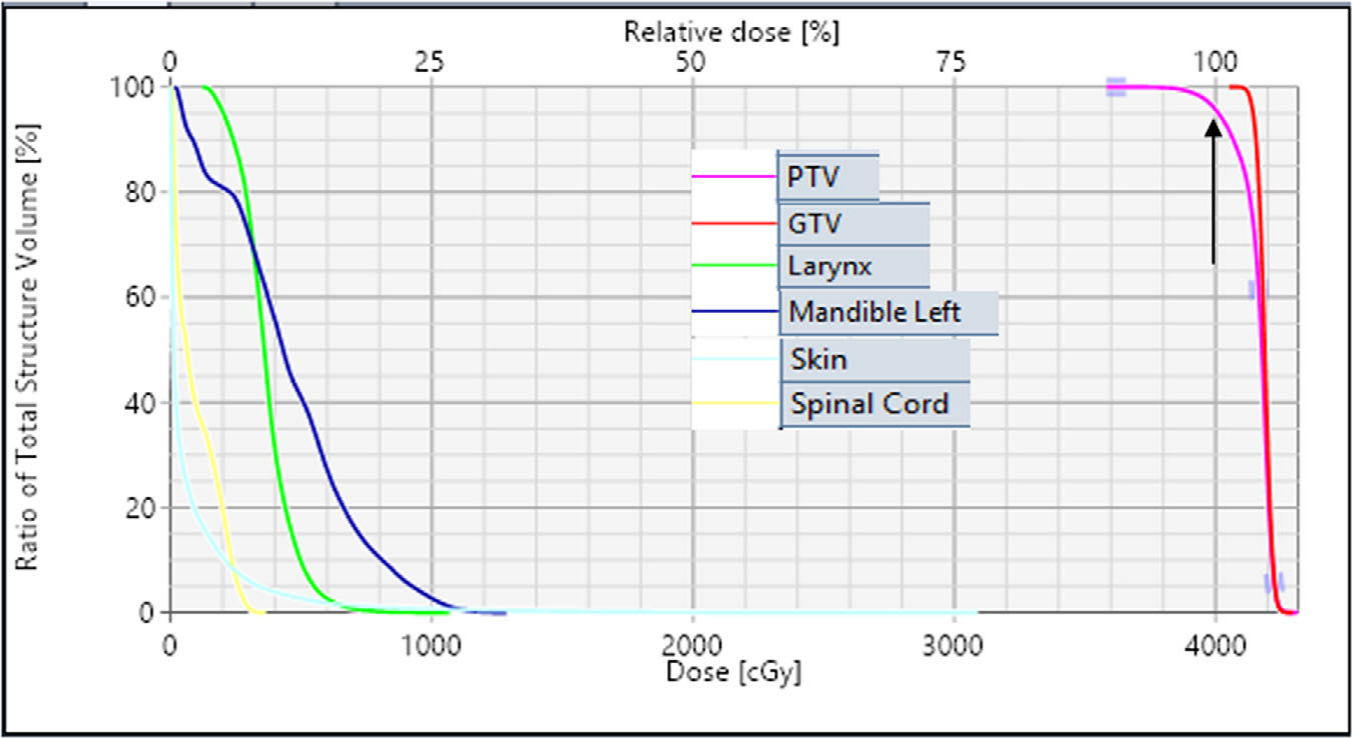
The dose–volume histogram (DVH) for the planning target volume (PTV) coverage (pink); 95% of the PTV received 100% of the prescription dose (black arrow) and greater than 100% of the minimum dose to the gross tumor volume (GTV, red). The organs at risk (OAR) sparing is shown for the larynx, mandible, skin, and spinal cord

For the clinical example case (patient # IV) shown in Figure 2, the PTV was 28.1 cm^3^ (3.8 cm diameter) and located in the left neck at the C4 level. The corresponding DVHs for the same patient is shown in Figure 3. The HyperArc VMAT SRT plan consisted of three non‐coplanar partial arcs. Using 40 Gy in five fractions treatment, 2497 MU per fraction was delivered. Beam on time was 3.12 min, and total treatment time (including patient set up, conebeam CT imaging time, and dry run) was within 15 min. In this case, the HyperArc VMAT plan provided highly conformal dose distribution. CI, PCN, *D*_2cm_ and GI were 1.05, 0.88, 35.6%, and 2.81, respectively, and all parameters were highly desirable for the SRT treatment. Maximal dose to OAR (0.03 cm^3^) including the spinal cord, left mandible, larynx, and skin were 3.3 Gy, 12.1 Gy, 8.3 Gy, and 27.5 Gy, respectively.

### 3.3 Target coverage and conformity

All HyperArc VMAT recurrent neck SRT plans demonstrated highly conformal PTV coverage, adequate dose to GTV, fast intermediate dose fall‐off with low values of gradient index and *D*_2cm_, as shown in Table 2. Overall, the minimum and mean doses to the GTV were 100% and 105% of the prescribed dose, respectively. The HyperArc VMAT plans exhibited RTOG CI and PCN values closer to unity, suggesting that the HyperArc method can produce highly conformal dose distributions. Small values of GI (< 3.0) and the maximum *D*_2cm_ away from the PTV in any direction (< 40.0) suggested that small intermediate dose‐spillage in the adjacent normal tissues with HyperArc VMAT plans which is highly desirable for re‐irradiation head and neck SRT.

**TABLE 2:** Summary of various treatment plan characteristics for all seven recurrent head and neck SRT patients treated with HyperArc VMAT. Dose was 30–40 Gy in five fractions. Mean ± SD (range) was reported. SD = standard deviation; PCN = Paddick conformation number; CI = Conformity index; HI = Heterogeneity index; GI = Gradient index. *D*_2cm_ = Maximal dose at 2 cm away from the PTV

**PTV**	**Volume covered by Rx dose (%)**	**96.3 ± 0.4 (95.6–96.6)**
	PCN	0.86 ± 0.04 (0.79–0.91)
	CI	1.03 ± 0.03 (0.99–1.07)
	HI	0.12 ± 0.04 (0.07–0.18)
Intermediate dose fall‐off	*D*_2cm_ (%)	34.93 ± 2.48 (31.40–39.30)
	GI	2.46 ± 0.41 (2.05–2.91)
Dose to GTV	Minimum (%)	99.9 ± 0.7 (98.9–101.9)
	Maximum (%)	112.3 ± 3.8 (107.5–118.4)
	Mean (%)	105.4 ± 2.3 (103.6–110.6)

### 3.4 Dose to adjacent OAR

For these previously irradiated head and neck SRT plans, clinically relevant dose to immediately adjacent OAR was maintained following the as low as possible principle. In this cohort, for each patient, the HyperArc VMAT SRT plan provided significantly low maximal dose to adjacent OAR: optic apparatus (< 4.8 Gy), brainstem (< 9.4 Gy), spinal cord (< 4.5 Gy), mandible (6.5 Gy), larynx (< 6.4 Gy), skin (23.4 Gy), and mean brain dose (< 2.2 Gy), respectively.

### 3.5 Treatment deliverability

Treatment delivery efficiency was documented by recording the total number of MU, beam MF, BOT, and overall treatment time during the patient treatment. For the different prescription dose, the HyperArc VMAT plans provided 2565 MU on average, corresponding to a relatively lower MF of 3.71 (Table 3). The major advantage of the HyperArc plans was faster treatment delivery with BOT and overall treatment time of less 3.5 min and 15 min, respectively. The pre‐treatment patient‐specific PD‐QA results and independent dose verification using an in‐house MC calculation suggested accurate delivery of the HyperArc VMAT SRT plans (Table 3). Independent dose verification using an in‐house MC calculation agreed with the planned dose distribution within 97.3%.

**TABLE 3:** Summary of treatment delivery parameters (SD and range) treated with HyperArc VMAT for all seven recurrent head and neck SRT patients. SD = standard deviation. MC = Monte Carlo

**Treatment delivery parameters**	
Total monitor units (MU)	2565 ± 521 (1722–3338)
Modulation factor (MF)	3.71 ± 0.89 (2.46–4.98)
BOT (min)	3.25 ± 0.65 (2.21–4.17)
Treatment time (min)	13.21 ± 0.65 (12.15–14.17)
Pre‐treatment PD–QA pass rate [2%/2 mm] (%)	98.7 ± 1.7 (95.5–99.9)
MC calculation, agreement (%)	97.3 ± 1.8 (96.7–100.0)

### 3.6 Patient set up and verification

**FIGURE 4:**
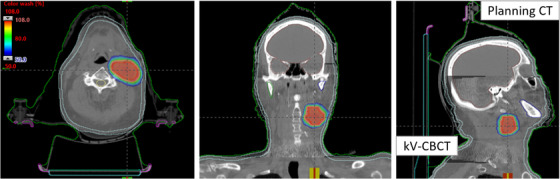
Overlay of the pre‐treatment kV‐CBCT with the planning CT images on the axial, coronal, and sagittal views of HyperArc VMAT SRT treatment of the same patient. In addition to anatomical landmarks, the planned dose colorwash (50%–108% isodose cloud) was overlaid at the treatment console for this treatment to demonstrate the delivery accuracy. The CBCT images were acquired in the treatment position and rigid‐registration was performed via automatic image‐registration with well‐defined region of interest followed by manually fine‐tuning the registration for better alignment of the target and spinal cord via soft‐tissue matching

Figure 4 shows the patient set up and verification on TrueBeam with pre‐treatment conebeam CT (CBCT) images of the same example patient #IV. The planned isodose color wash was superimposed with the daily CBCT images after the six degrees‐of‐freedom (6DoF) couch corrections were applied. This patient was initially positioned using external marks drawn on the Qfix mask and lined up with the in‐room lasers, followed by the isocenter shifts and pre‐treatment CBCT scan being obtained.

An in‐house SRT/IGRT protocol was applied registering the pre‐treatment CBCT images with the planning CT scans (see Figure 4). Rigid‐registration was performed automatically based on region of interest and bony landmarks followed by a manual refinement of the soft‐tissues matching and confirmed by the treating physician and physicist for the alignment of target and spinal cord. The patient was then repositioned by applying the 6DoF couch corrections from the original isocenter and the treatment was delivered. Our Linac‐based image‐guided SRT/IGRT protocol limits translational, and rotational couch corrections to less than ±2.0 mm and 2° in each direction, respectively, for all HyperArc SRT treatments.

## 4 DISCUSSION

A novel application of highly non‐coplanar HyperArc VMAT geometry in the treatment of recurrent head and neck tumors using SRT is shown. Highly conformal dose distributions and OAR sparing were shown via independent dose verification, in‐house end‐to‐end phantom tests and the wide range of clinical patient's plans down to the C4 vertebral level. Due to the proximity of the many dose limiting organs near the recurrent target in the head and neck region, highly conformal dose distributions are greatly desirable to reduce the chances of radiation induced toxicity. HyperArc VMAT SRT plans were highly conformal and achieved adequate target coverage (see Table 2 for dose to GTV, CI, PCN) with fast intermediate dose‐fall off. For all patients, the HyperArc VMAT plans provided low maximal dose to the adjacent OAR, including sparing the spinal cord, for these re‐treatment patients. Due to the faster patient set up with the Encompass device, fast set‐up verification, and treatment delivery workflow, the HyperArc VMAT treatment was well tolerated by all the patients. The average overall treatment time (door‐to‐door) was less than 15 min per treatment. The HyperArc VMAT‐SRT portal‐dosimetry QA gamma passing rates of 98.7%, on average demonstrated an excellent potential for fast, reliable, and accurate delivery using head and neck SRT treatment for recurrent tumors.

For highly conformal dose distribution, Rwigema et al. showed the incorporation of non‐coplanar IMRT fields in head and neck SBRT with the 4π radiotherapy technology approach could potentially further limits the dose to OAR.^28^ For 27 patients with 29 tumors (35–44 Gy in 5 fractions), they have demonstrated mean dose reductions of > 50% to the spinal cord, brainstem, parotids, and larynx, as well as significant reductions in the 50% isodose spillage volume and the probability of late toxicities. Although, the dosimetric advantages, safety and feasibility of 4π delivery approach was reported in a follow‐up prospective clinical trial for recurrent high‐grade glioma patients;^29^ the 4π algorithm used up to 30 highly optimized coplanar/non‐coplanar IMRT fields, significantly prolonging overall treatment time. Moreover, in these reports total MU and treatment delivery time using the 4π delivery was not reported. As of now, the 4π delivery algorithm is not clinically available, and delivering 30 c/n‐coplanar IMRT fields to treat head and neck SRT patients would be clinically impracticable (for the current clinical Linacs) due to potential collision issue and the therapists need to enter the treatment room many times. Utilizing HyperArc VMAT overcomes these concerns. We have demonstrated that the fully automated non‐coplanar HyperArc VMAT allows to deliver head and neck SRT treatments within 15 min without the patient collision issue (with virtual dry run during treatment planning) and no need for therapists to enter the treatment room, compared to 4π algorithm (presumably very long), or robotic CyberKnife (30–120 min).^8^ By significantly shortening the overall treatment time, the risk of deviating from the planned dose delivery is decreased as the patient is less likely to move and thus improve the patient comfort and clinical workflow.

Recently, there were two retrospective studies published in the treatment of head and neck SRT using HyperArc VMAT planning.^30^, ^31^ Ho et al. reported their dosimetric comparison study of HyperArc approach over the manual coplanar RapidArc geometry.^30^ Ten head and neck patients with only recurrent nasopharyngeal carcinoma were replanned with HyperArc geometry for a total dose of 36.75 Gy in 5 fractions. In this cohort, they have demonstrated better target conformity, faster intermediate‐dose fall‐off with HyperArc plans but no significant reduction of dose to OAR. Another retrospective study by Woods et al. from UCLA Medical Center demonstrated the potential for dose escalation utilizing non‐coplanar HyperArc VMAT geometry in the treatment of recurrent head and neck cancer patients.^31^ In their study, 20 previously treated head and neck cancers patients with a total dose of 40 Gy in 5 fractions were re‐planned utilizing HyperArc technique with escalated tumor dose of as high as possible, while maintaining the effective dose to the OAR. Their results suggested that, for similar effective OAR doses, the mean PTV dose and GTV dose can be escalated by 10.8 Gy (25%) and 11.5 Gy (26%), on average, respectively—potentially enabling improved tumor local control rate for recurrent head and neck cancer patients without increased risk of treatment related toxicity. These early two retrospective papers prepared the foundation for the clinical implementation of head and neck SRT using HyperArc VMAT. In contrast to these studies, in our prospective clinical study we have demonstrated actual patient's treatment using HyperArc geometry after the clinical validation and independent dose verification using MD Anderson's SRS head phantom. Moreover, 13 out of 20 plans presented by Woods et al.^31^ manually adjusted their isocenter location off‐center due to the concern of risk of collision issue, that would be localized outside the patient‐specific protection zone; in contrast to their retrospective study, we have validated our prospective treatment planning approach and automatic localization of the treatment isocenter within the C4 vertebral level in the head and neck target, with no concerns of patient collision issue while using the patient protection zone that eliminates the risk of gantry collision with the patient.

In summary, we have demonstrated highly conformal target coverage, sharp dose fall‐off (50% fall‐off within 5 mm from the surface of the target volume) and significantly sparing adjacent critical organs including the spinal cord (< 5 Gy). Perfect pitch couch corrections from the skin markers was on average <1.5 mm and 2° in each direction on average, with overall treatment completed within 15 min. The caveat of this study is the limited number of patients treated. However, we are continuing to treat recurrent head and neck SRT patients with HyperArc VMAT geometry, and collecting clinical data in for a clinical follow‐up study. Future research includes analyzing rotational correction errors and intra‐fraction movement errors including further validation of continuous patient couch rotation during HyperArc VMAT treatment delivery.^32^, ^33^ Clinical follow up of these patients is ongoing to confirm the therapeutic benefits of this novel and attractive head and neck SRT treatment.

## 5 CONCLUSION

HyperArc VMAT provided highly conformal dose distribution, rapid dose fall‐off, excellent sparing of adjacent critical organs, highly precise and accurate treatment that could be delivered to as low as the C4 vertebral level within 15 min door‐to‐door time. It could potentially allow HyperArc SRS/SRT to treat jugular glomus tumors or for patients who may not tolerate the frame‐based SRS treatment. For clinics equipped with HyperArc VMAT module, fast and effective treatment of recurrent head and neck SRT patients is possible and recommended.

## FUNDING

None.

## AUTHOR CONTRIBUTIONS

Damodar Pokhrel and Mahesh Kudrimoti conceptualized the project. Damodar Pokhrel collected and analyzed the data for commissioning and clinical implementation of the HyperArc VMAT SRT and drafted the preliminary manuscript. Mahesh Kudrimoti, Mark E. Bernard, Denise Fabian, and Jeremiah Johnson provided clinical expertise and supervision of the paper. All co‐authors revised and approved the final manuscript.

## CONFLICT OF INTEREST

The authors declare that there is no conflict of interest that could be perceived as prejudicing the impartiality of the research reported.

## Data Availability

Research data are not shared.
